# Folate-Receptor Positive Circulating Tumor Cell Is a Potential Diagnostic Marker of Prostate Cancer

**DOI:** 10.3389/fonc.2021.708214

**Published:** 2021-10-08

**Authors:** Shenyi Lian, Lujing Yang, Qin Feng, Ping Wang, Yue Wang, Zhongwu Li

**Affiliations:** Key Laboratory of Carcinogenesis and Translational Research (Ministry of Education/Beijing), Department of Pathology, Peking University Cancer Hospital & Institute, Beijing, China

**Keywords:** circulating tumor cells, prostate cancer, folate-receptor positive, diagnosis, biomarkers

## Abstract

Folate-receptor positive circulating tumor cells (FR+CTCs) shows an important role in the diagnosis and dynamic monitoring for many solid tumors; however, the application of FR+CTCs in prostate cancer remains unclear. We explored the potential application of FR+CTCs in this retrospective study. The levels of FR+CTCs were detected in 30 prostate cancer patients and 7 bladder cancer patients in Peking University Cancer Hospital from August 2017 to August 2021. Clinical and pathology data were collected. One-way ANOVA was used to compare the difference in FR+CTCs levels in patients with prostate cancer, bladder cancer, and benign disease. The area under the receiver operating curve (AUROC) was used to compare the accuracy of FR+CTCs and tPSA in the diagnosis of prostate cancer. We found that levels of FR+CTCs were significantly higher in cancer patients (both prostate and bladder cancer) than in patients with benign urinary disease (*p* < 0.001). Besides, FR+CTCs level was consistently high in the prostate cancer patients with different tPSA levels (*p* < 0.001), and it was significantly higher in the patients with f/tPSA levels <0.16 than in those patients with f/tPSA levels >0.16 (12.20 ± 1.31 vs. 8.73 ± 0.92 FU/3 ml, *p* = 0.043). The diagnosis efficiency of FR+CTCs is better than the tPSA in prostate cancer patients with tPSA <10 ng/ml (0.871 vs. 0.857). In the prostate cancer patients with tPSA <10 ng/ml and f/tPSA <0.16, a combination of FR+CTCs and tPSA (AUROC, 0.934) further increased the diagnosis efficiency of each of these biomarkers alone (FR+CTCs, 0.912; tPSA, 0.857). Therefore, FR+CTCs could serve as an early diagnosis marker in the prostate cancer patients with uncertain tPSA levels.

## Introduction

Prostate cancer is the second common male malignant in the global. The incidence of prostate cancers in China is consistently increasing in recent years. The patients whose serum tPSA level >4.0 ng/ml should be subject to transrectal ultrasound-guided prostate biopsy (TRUS-Bx) (1). The positive ratio of initial TRUS-Bx for the men with elevated tPSA is only 40–45% (2, 3). A systematic meta-analysis of prostate cancer in China indicated that the sensitivity and specificity threshold of tPSA for prostate cancers candidates were heterogeneous depending on the level of tPSA (4). tPSA alone, especially when tPSA <10 ng/ml, is not effective for the diagnosis of prostate cancers [summary receiver operating characteristics (SROC), 80%]. The combination of f/tPSA and prostate volume increased the diagnosis power in men without clinical detectable PCs (5).

Liquid biopsy has been a recently developed non-invasive approach for early diagnosis and dynamic monitoring of the malignant disease in many solid tumors (5–9). CTCs are the tumor-like cells shed from the primary and metastasis tumor sites and enter into the circulating system. Several pilot studies indicated that liquid biopsy becomes a diagnostic and prognostic biomarker in solid tumors, which would potentially challenge the status of tissue biopsy, especially for the metastatic patients who were intolerant of tissue biopsy (5–9). Liquid biopsy is a non-invasive method to analyze the characteristics of primary or metastasis sites by collecting the circulating tumor cell, cell-free DNA (ctDNA), and other bioliquids (urine or cerebrospinal fluid). CELLSEARCH system was used as a “gold standard” reference in the CTCs detection (10). Recent studies reported that 7.5 ml blood in CELLSEARCH system is too little to reliably determine the tumor heterogeneity by staining the CD45/CK/VIM (11). For a subset of prostate cancers patients, there were no correlations between presurgical CTCs numbers and biochemical recurrence (12, 13). Therefore, the development and evaluation of other CTCs detection methods for urinary malignance is urgent and important.

CytoploRare detection kits utilized negative enrichment and ligand-target PCR (LT-PCR) to relatively quantify the CTCs in the peripheral blood (14, 15). In the peripheral blood, only a small fraction of cells expressing the folate receptor (FR) including CTCs and a subtype of activated monocytes are usually detected in the malignant disease. Folate receptor, especially FRα; is a glycoprotein highly expressed on the cell membrane of a spectrum of solid tumors, including ovarian, kidney, breast, lung, colorectal, prostate, testicular, bladder, and non-small cell lung cancers (16). Therefore, the synthesized oligonucleotide conjugated with FRα is a suitable bait for capturing the CTCs (17, 18). Several studies have reported the applications of FR+CTCs in lung cancer, pancreatic cancer, and urinary cancer (5–7, 18, 19). The dynamic change in FR+CTCs level can also predict the outcome of EGFR-TKi and chemotherapy treatment of NSCLC patients (7).

The utility of FR+CTCs in prostate cancer has not been systematically studied. In this study, we investigated the potential diagnostic significance of FR+CTCs in prostate cancer patients, especially those patients with uncertain tPSA levels.

## Materials and Methods

### Patients

In this study, 30 newly diagnosed, treatment-naive sporadic prostate cancer patients were enrolled during August 2017 and August 2021. Diagnosis of prostate cancer was based on the pathological observation of the needle biopsy or surgical resection. We enrolled the patients into prostate cancer group following these rules: the patients have been suspected of prostate cancer with elevated tPSA or clinical manifestation; the FR+CTCs detection should be taken before the TRUS-Bx biopsy; and the date of tPSA and f/tPSA examination should be closer to the date of the FR+CTCs detections (± 2 days). Clinical data including age, tumor stage, Gleason score, WHO grading, vascular infiltration, tPSA (0–4 ng/ml), f/tPSA (0.16–100), and the positive rate of needle biopsy were collected. In addition, seven patients with the benign disease were also included. The patients’ characteristics are listed in **Table 1**. The study was approved by the Ethics Committee of Peking University Cancer Hospital.

**Table 1:** Patient characteristics.

Characteristics	No. of patients
**Prostate cancer (n = 30)**	
Age	
≤65	16 (53.3%)
>65	14 (46.7%)
Tumor stage	
T2	14 (46.7%)
T3	16 (53.3%)
Gleason grading	
1/2	12 (40.0%)
3/4/5	18 (60.0%)
Prognostic grading	
1/2b	10 (33.3%)
3a/3b/3c	20 (66.7%)
tPSA	
>10 ng/ml	15 (50.0%)
<10 ng/ml	15 (50.0%)
fPSA/tPSA	
<0.16	26 (86.7%)
≥0.16	4 (13.3%)
Vascular invasion	
Present	4 (13.3%)
Absent	26 (86.7%)
Lymph node metastasis	
Absent	28 (93.3%)
Present	2 (6.7%)
Biopsy positive rate	
>60%	5 (16.7%)
≤60%	18 (60.0%)
N/A	7
**Bladder cancer (n = 7)**	
Age	
≤60	2 (28.6%)
>60	5 (71.4%)
Histopathological type	
Invasive	3 (42.9%)
Non-invasive	4 (57.1%)
Tumor differentiation	
Well	3 (42.9%)
Poor	4 (57.1%)
Vascular invasion	
Present	2 (28.6%)
Absent	5 (71.4%)
**Benign diseases (n = 7)**	
Age, year (range)	
Renal calculus	3 (42.9%)
Prostatitis	4 (57.1%)

### Circulating Tumor Cell Detection

FR+CTCs were determined by CytoploRare Kit (GenoSaber Biotech Co. Ltd, Nantong, China), a commercial kit approved by China Food and Drug Administration (FDA). In brief, the method was divided into two major steps: CTC-negative enrichment through immunomagnetic beads and CTC quantification by the ligand target PCR. Three milliliters of whole blood sample was collected into anticoagulant tubes. Samples were analyzed strictly according to the manufacturer’s protocol within 12 h. Briefly, erythrocytes in whole blood were lysed, and leukocytes were depleted by anti-CD45 immunomagnetic beads. Next, FR+CTCs were labeled by the probe conjugated with folic acid and synthesized oligonucleotide at room temperature for 40 min. The probe containing the FRα folic acid unit was designed for specific binding with FR-positive cells. It also has an oligonucleotide unit (5′-CTCAA CTGGT GTCGT GGAGT CGGCA ATTCA GTTGA GGGTT CTAA-3′) for the semiquantification PCR. The unbound probes were washed off. Fluorescence quantitative PCR was run on the ABI 7500 instrument (Thermo Fisher, Waltham, MA, USA). The amplification cycle was set as follows: 95°C denaturation for 2 min, 40°C annealing for 30 s, 60°C extension for 1 min, 8°C cooling for 5 min, followed by denaturation at 95°C for 1 min and 40 cycles of denaturation at 95°C for 10 s, annealing at 35°C for 30 s, and extension at 72°C for 5 s. The primer sequences were as follows: forward primer, 5′-TATGA TTATG AGGCA TGA-3′; reverse primer, 5′-GGTGT CGTGG AGTCG-3′; the TaqMan probe, 5′-FAM-CAGTT GAGGG TTC-MGB-3′. The oligonucleotides were analyzed by quantitative PCR with a serial of standards containing oligonucleotides ranging from 10^−14^ to 10^−9^ M and used for FR+CTCs quantification, which represents the 2–2 × 10^5^ FU/3 ml blood. “FU” was defined as FR+CTCs numbers.

### Total PSA and f/t PSA Measurement

Total PSA and free PSA levels in peripheral blood were measured by electrochemiluminescence assay (Roche). The reference ranges of total PSA and f/t PSA were 0–4 U/ml and 0.16, respectively.

### Statistical Analysis

We focused on the comparison of FR+CTCs levels in prostate cancer and benign disease. The primary endpoint of this study is the difference in FR+CTCs levels in the cancer patients and the benign disease patients. The second endpoint is the diagnosis performance of FR+CTCs as a biomarker for the diagnosis in prostate cancer with uncertain tPSA level. For this purpose, the calculated sample size of malignant disease is 17 (β = 0.90, α = 0.01). Sample size was calculated by the PASS 15.0.10.

Data were analyzed by the IBM SPSS, v.21(IBM Corp.), and the graphs were processed by the PRISM 5 (GraphPad, Inc.). FR+CTCs were summarized as mean ± standard error. To compare the FR+CTCs between different groups, we used the Student’s t-test and ANOVA test. Receiver operating characteristic (ROC) curves were used to determine threshold associated with high sensitivity and specificity, and the area under ROC curve (AUROC) was calculated by the FR+CTCs, tPSA, and f/tPSA index. *p* < 0.05 was considered significantly different. The statistical analysis was supervised by Prof. Zhonghu He.

## Results

### The FR+CTCs Levels Are Higher in Patients With Urinary Tract Cancer Than in Patients With the Benign Disease

**Figure 1:**
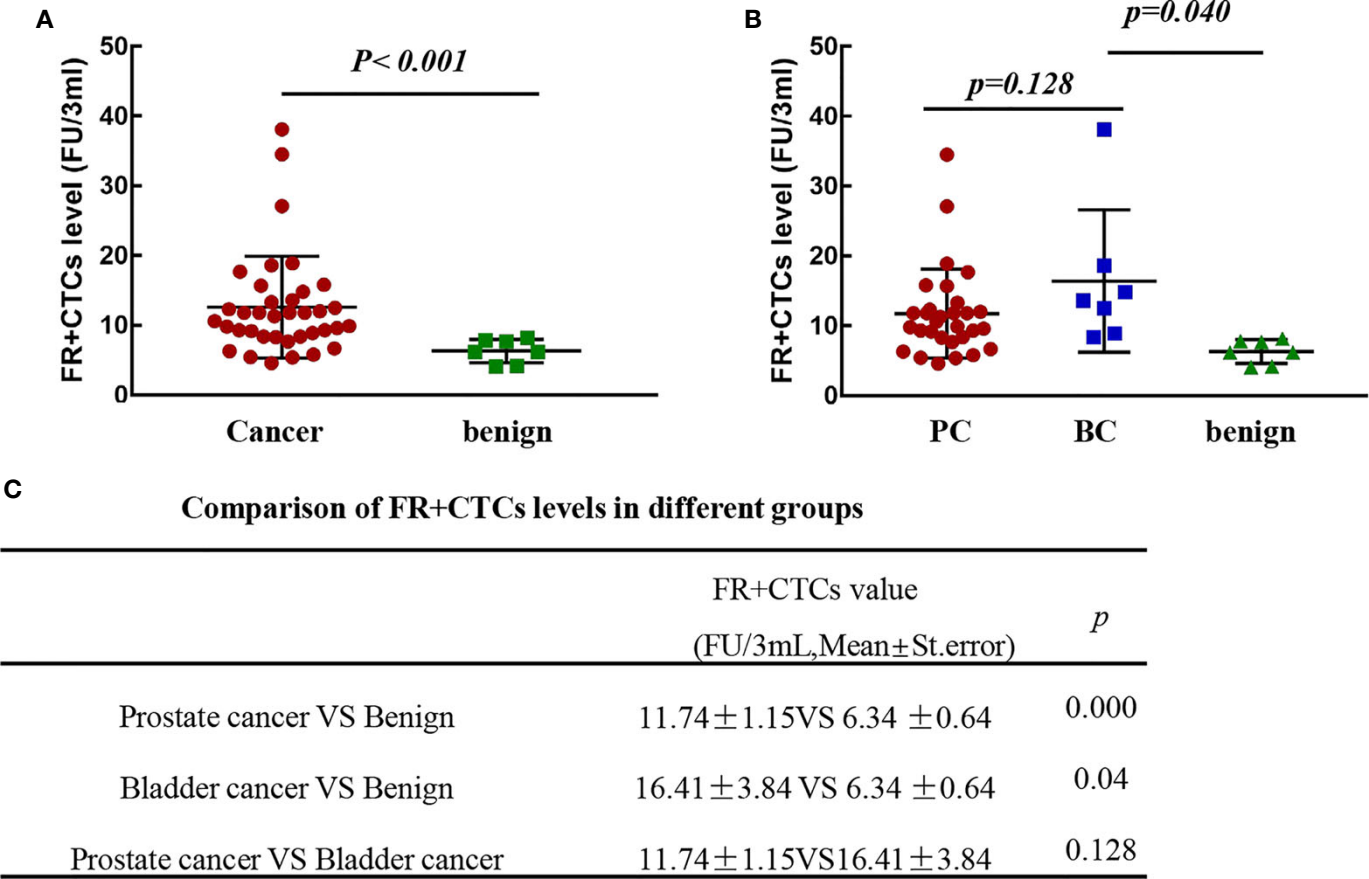
The FR+CTCs levels in patients with the urinary malignant and benign diseases. **(A)** Comparison of FR+CTCs levels in patients with malignant and benign diseases. **(B, C)** Comparison of FR+CTCs levels in patients with prostate cancer (PC), bladder cancer (BC), and benign disease.

To evaluate the potential applications of FR+CTCs in the urinary tract cancers, we retrospectively collected the clinical data from 37 malignant patients (prostate cancers, n = 30; 7 bladder cancers, n = 7) and 7 benign disease patients (renal calculus, n = 3; prostatitis, n = 4). The FR+CTCs level in the peripheral blood is presented as means and standard error (mean ± standard error). The FR+CTCs levels of the 37 malignant patients were significantly higher than those of the benign disease patients (12.62 ± 1.20 vs. 6.34 ± 0.64 FU/3 ml, *p* < 0.001) (**Figure 1A**). The FR+CTCs levels of bladder cancer patients were also markedly higher than those of the benign patients (16.41 ± 3.84 vs. 6.34 ± 0.64 FU/3 ml, *p* = 0.040) (**Figures 1B, C** **)**. However, no significant difference between the two malignant diseases was observed (11.74 ± 1.15 vs. 16.41 ± 3.84 FU/3 ml, *p* = 0.128) (**Figures 1B, C** **)**.

### The Association of FR+CTCs Levels With Clinic–Pathological Characteristics in Prostate Cancer

To further explore the application of FR+CTCs for prostate cancer, we analyzed the correlations between FR+CTCs and clinical pathological characteristics. All the information of sporadic prostate cancer groups is summarized in **Table 2**. None of the patients has been received anticancer therapy before being diagnosed with prostate cancer. The median age of prostate cancer patients was 65.0 years (range, 45–80 years). Among them, 14/30 (46.7%) patients were in T2 stage, and 16/30 (53.3%) were in T3 stage. Gleason grade and prognostic grade were identified by the Gleason score and tumor–node–metastasis (TNM) stage according to the NCCN guideline (2020.V2). There was no significant correlation between the FR+CTCs levels and the age, the Gleason grading, and the prognostic stage. Although there was no difference in FR+CTCs levels in the patients with different tPSA levels, we found that the FR+CTCs levels were significantly higher in the patients with f/tPSA <0.16 (12.20 ± 1.31 vs. 8.73 ± 0.92 FU/3 ml, *p* = 0.043, **Table 2**) than in patients with f/tPSA >0.16. The f/tPSA value played an important role in the diagnosis of prostate cancer with tPSA <10 ng/ml. The lower the f/tPSA value, the higher the risk of prostate cancer. Therefore, FR+CTCs may serve as a potential biomarker of the patients with uncertain tPSA levels.

**Table 2:** Correlation of FR+CTCs levels and clinical characteristics in prostate cancer.

Characteristics	No. of patients	FR+CTC(FU/3 ml, means ± standard error)	*p*
Prostate cancer	30		
Age			*p* = 0.257
≤65	16 (53.3%)	10.36 ± 1.10	
>65	14 (46.7%)	12.94 ± 1.93	
Tumor stage			*p* = 0.579
T2	14 (46.7%)	11.043 ± 1.1.5	
T3	16 (53.3%)	12.35 ± 1.73	
Gleason grading			*p* = 0.276
1/2	12 (40.0%)	10.20 ± 1.70	
3/4/5	18 (60.0%)	12.76 ± 1.56	
Prognostic grading			*p* = 0.452
1/2b	10 (33.3%)	10.48 ± 1.99	
3a/3b/3c	20 (66.7%)	12.37 ± 1.44	
tPSA			*p* = 0.868
>10 ng/ml	15 (50.0%)	11.94 ± 1.87	
<10 ng/ml	15 (50.0%)	11.54 ± 1.43	
fPSA/tPSA			*p = 0.043*
<0.16	26 (86.7%)	12.20 ± 1.31	
≥0.16	4 (13.3%)	8.73 ± 0.92	
Vascular invasion			*p* = 0.441
Present	4 (13.3%)	13.45 ± 2.01	
Absent	26 (86.7%)	11.48 ± 1.30	
Lymph node metastasis			*p* = 0.961
absent	28 (93.3%)	11.73 ± 1.24	
Present	2 (6.7%)	11.80 ± 0.50	
Biopsy positive rate			*p* = 0.067
>60%	5 (16.7%)	14.26 ± 1.68	
≤60%	18 (60.0%)	9.95 ± 1.18	
N/A	7		

### The FR+CTCs Levels Was Higher in Prostate Cancers With the Suspected tPSA and f/tPSA

**Figure 2:**
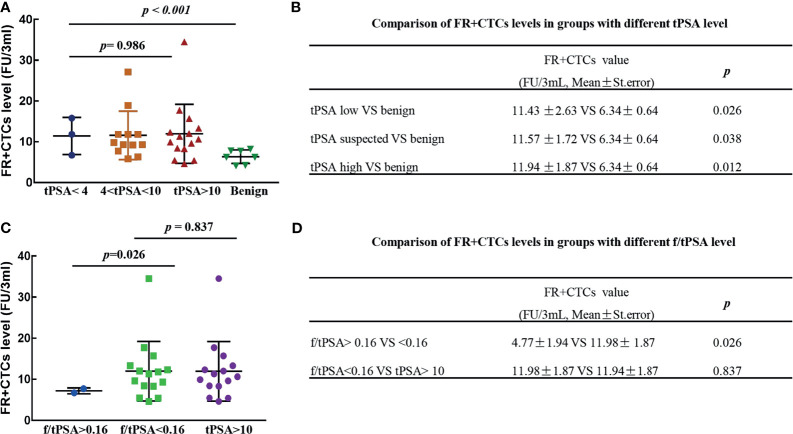
The FR+CTCs levels in prostate cancer with different tPSA levels. **(A, B)** Comparison of FR+CTCs levels in patients with tPSA < 4 ng/ml, 4 ng/ml < tPSA < 10 ng/ml, tPSA > 10 ng/ml. **(C, D)** Comparison of FR+CTCs levels in patients with different f/tPSA levels.

To further verify the role of FR+CTCs in the prostate cancers with uncertain tPSA level, we divided the prostate cancer patients into three groups. Patients with tPSA level <4 ng/ml were assigned as tPSA-low group, patients with tPSA level between 4 and 10 ng/ml as tPSA-suspected group, and patients with tPSA level >10 ng/ml were assigned as the tPSA-high group. The average level of FR+CTCs in the tPSA-low group is higher than in the benign group (11.43 ± 2.63 vs. 6.34 ± 0.64 FU/3 ml, *p* = 0.026), the level FR+CTCs in the tPSA-suspected group was also higher than that in the benign group (11.57 ± 1.72 vs. 6.34 ± 0.64 FU/3 ml, *p* = 0.038). There was no significant difference in FR+CTCs levels between the tPSA-low, tPSA-suspected, and tPSA-high group (*p* = 0.986) (**Figures 2A, B** **)**. We further focused on the patients with tPSA levels lower than 10 ng/ml. There were two groups classified by the f/tPSA with cutoff value of 0.16. The FR+CTCs levels in patients with f/tPSA lower than 0.16 was significantly higher than in the patients with f/tPSA higher than 0.16. (11.98 ± 1.87 vs. 4.77 ± 1.94 FU/3 ml, *p* = 0.026, **Figures 2C, D** **)**. There was no difference in the FR+CTCs in the prostate cancer patients with f/tPSA <0.16 and tPSA >10 ng/ml patients (11.98 ± 1.87 vs. 11.94 ± 1.87 FU/3 ml, *p* = 0.837, **Figures 2C, D** **)**. These results indicated that FR+CTCs detection is helpful in the diagnosis of patients with tPSA <10 ng/ml.

### The Diagnosis Efficiency of FR+CTCs in the Prostate Cancer

**Figure 3:**
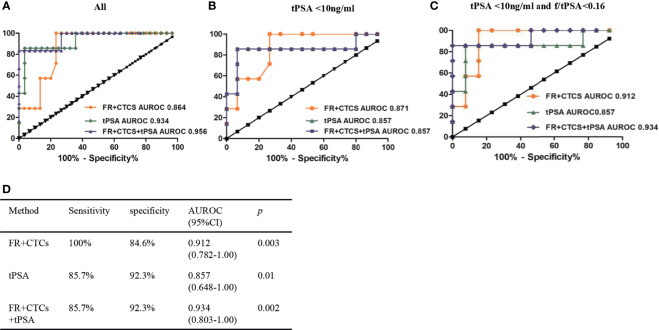
ROC curve of FR+CTCs, tPSA, and the combination for the prostate cancer. **(A)** ROC curve of FR+CTCs and tPSA in all prostate cancer. **(B)** ROC curve of FR+CTCs, tPSA, and their combination in the prostate cancer with tPSA <10 ng/ml. **(C)** ROC curve of FR+CTCs, tPSA, and their combination in the prostate cancer with tPSA <10 ng/ml and f/tPSA <0.16. **(D)** The diagnostic efficiencies of FR+CTCs, tPSA, and the combination in patients with tPSA <10 ng/ml and f/tPSA <0.16.

Next, we further compared the diagnosis efficiency of FR+CTCs and tPSA in the prostate cancer. In all the prostate cancer patients, the AUROC of FR+CTCs was 0.864 (95% CI, 0.745–0.983, *p* = 0.003), and the optimal cutoff value is 8.25, with a sensitivity of 100% and a specificity of 76.7%. The AUROC of tPSA is 0.934 (95% CI, 0.831–1.00, *p* = 0.001) (**Figure 3A**). To further evaluate the potential diagnosis efficiency of FR+CTCs in the suspected prostate patients, we first calculated the AUROC of FR+CTCs and tPSA in the patients with tPSA <10 ng/ml. In this group (n = 15), the diagnosis efficiency of FR+CTCs (0.871; 95% CI, 0.724–1.00; *p* = 0.006) was better than tPSA (0.857; 95% CI, 0.644–1.00; *p* = 0.008) (**Figure 3B**). When we combined the FR+CTCs and tPSA, the diagnosis efficiency of the combination (AUROC, 0.857; 95% CI, 0.644–1.00; *p* = 0.008) was poorer than that of FR+CTCs alone; it did not change the diagnosis efficiency of tPSA alone. Then, we calculated the AUROC in the patients with tPSA <10 ng/ml and f/tPSA <0.16 (n = 13). The AUROC of FR+CTCs in this group was higher than that of tPSA (0.912; 95% CI, 0.782–1.00, *p* = 0.003 vs. 0.857; 95% CI, 0.648–1.00, *p* = 0.010). The combination of FR+CTCs and tPSA (AUROC, 0.934; 95% CI, 0.803–1.00; *p* = 0.002) further increased the diagnosis efficiency of each alone (**Figures 3C, D**). Therefore, the FR+CTCs detection could be a powerful diagnosis method for prostate cancer patients with uncertain tPSA and f/tPSA.

## Discussion

FRα has been applied in the diagnosis and treatment of cancer for many decades, such as FRα target radionuclide contrast agent, folate–drug conjugate, and FRα–fluorescein isothiocyanate (FITC) conjugate probes in surgery (20). CytoploRare Kit was the first liquid biopsy kit designed base on the overexpression of FRα in solid tumors. The application of FR+CTCs using CytoploRare Kit has been identified in non-small cell lung cancer, pancreatic cancer, and breast cancer (5–7, 18, 19). Recently, a multicenter prospective study released that the isolation CTC by size of epithelial tumor cell technique (ISET) is not suitable for lung cancer screening (21). CTC detection by FRα exhibited an excellent performance in the dynamic monitor in the EGFR-TKI treatment and pemetrexed-based chemotherapy in NSCLC (6, 7). Therefore, the application of FR+CTCs in other tumor types such as urinary system cancer needs to be explored.

In this study, we found that the FR+CTCs levels of malignant diseases (prostate cancer and bladder cancers) were significantly higher than in the benign diseases (*p* < 0.001). These results agreed with the diagnosis potential of FR+CTCs in solid tumors, which had been published by other groups (14, 18, 19, 22). Because the prostate cancer is the kind of “benign” tumor with insidious symptoms and longer survival times, we did not include the T1 or T0 patients in our retrospective study. According to the consistent high FR+CTCs levels in the prostate cancers with different tPSA status (**Figure 2**), we supposed that FR+CTCs elevated earlier and maintained higher stability than the other traditional biomarkers (tPSA or f/tPSA) during the development of the disease. Due to the lower incidence of prostate cancer in China, the small sample sizes of this study maybe the reason for the negative results of the correlation between FR+CTCs and clinical characteristics (T stage, Gleason grading, and prognostic grading) (23, 24). The pathological diagnosis following the TRUS-Bx is the gold diagnosis standard of patients with suspicious tPSA levels (4–10 ng/ml). Parts of prostate cancers patients was reluctant to the invasive operation when the tPSA or f/tPSA were in the “gray area” (4 ng/ml < tPSA < 10 ng/ml). We noticed that the level of FR+CTCs was significantly higher in the patients with f/tPSA <0.16 (12.20 ± 1.31 vs 8.73 ± 0.92 FU/3 ml, *p* = 0.043). There were no significant correlations with the level of FR+CTCs and TRUS-Bx biopsy positive rate; however, the FR+CTCs level was higher in the group of patients with >50% biopsy positive rate than those with <50% biopsy positive rate (14.26 ± 1.68 vs. 9.95 ± 1.18 U/3 ml, *p* = 0.067) (**Table 2**). It indicated that the FR+CTCs level could predict the result of TRUS-Bx and associated with the tumor accumulation and distribution in prostate organ. Therefore, detection of FR+CTCs should be considered in the prostate cancer patients with tPSA <10 ng/ml and f/tPSA <0.16 before the invasive TRUS-Bx operation.

tPSA was a golden biomarker in the prostate cancers for decades. We compared the diagnosis efficiency of FR+CTCs and tPSA. In the whole cohort, the AUROC of FR+CTCs was poorer than that of the tPSA (0.864 vs. 0.956, **Figure 3A**), but the AUROC of FR+CTCs was higher than that of the tPSA in the patients with tPSA <10 ng/ml. The combination of these two biomarkers further increased the diagnostic efficiency in the patients with tPSA <10 ng/ml and f/tPSA <0.16 (**Figures 3C, D**). These results indicated that the FR+CTCs maybe a more sensitive biomarker in the patients with uncertain tPSA levels.

Although we obtained encouraging results of FR+CTCs in the diagnosis of prostate cancers, this retrospective study also had three unavoidable limitations. The enrolled sample number was rather limited. Although we calculated the sample size before collecting patients, collection more cancer samples or benign patients would be better for the diagnosis comparison. The FR+CTCs detection kit was proved by China FDA in 2017. There were few FR+CTCs studies in the prostate cancer field; thus, the clinic doctors did not thoroughly acknowledge the powerful application of FR+CTCs in the diagnosis of prostate cancer. The clinical practices were almost halted from February 2020 to February 2021 because of the worldwide coronavirus disease 2019 (COVID-19). Therefore, FR+CTCs detections was not the routine examination in the cancer patients, especially in prostate cancer patients. The strict enrollment rules further decreased the sample size in our study. The second limitation was that the prognosis information, such as disease-free survival (DFS) and overall survival (OS), was absent in this study, and we did not evaluate the predictive role of FR+CTCs on prognosis in prostate cancer. The lower preoperative FR+CTCs predicted the longer elapse-free survival (RFS) and overall survival (OS) in non-small cell lung cancer (25). Another group found that high baseline FR+CTCs levels were associated with shorter PFS and OS after pemetrexed-based chemotherapy in non-squamous non-small cell lung cancer (6). The prognostic role of FR+CTCs in prostate cancer would be discussed and explored further. Third, dynamic monitoring of FR+CTCs in prostate cancers remained to be performed. Presently, we are trying to collect the serial FR+CTCs data of patients in different timepoints, such as before/after operation and before/after neo-immunotherapy. Until now, the preliminary data show that the FR+CTCs level in 50% of prostate cancer patients were decreased dramatically after the surgery, 25% of patients exhibited no change, while 25% of patients showed elevated FR+CTCs level after surgery (data not shown). We assumed that the different changes in FR+CTCs in prostate patients maybe associate with overall survival or disease-free survival. If the limitation mentioned above could be solved in further multiple center prospective studies, we believe that the role of FR+CTCs in early diagnosis and prognosis would be explored, and FR+CTCs could also be a biomarker in the dynamic monitoring for the prostate cancers.

## Data Availability Statement

The original contributions presented in the study are included in the article. Further inquiries can be directed to the corresponding author.

## Author Contributions

All authors participated in the design, interpretation of the studies and analysis of the data, and review of the manuscript. SL and ZL conceived and designed the study. SL, LY, and ZL contributed to the writing of the manuscript. QF, PW, and YW collected and analyzed the data. PW and YW performed FR+CTCs examinations. All authors contributed to the article and approved the submitted version.

## Funding

This research was supported by the Natural Science Foundation of China (grant number 81872309) and Beijing Hospitals Authority Youth Programme (grant number QML20191109).

## Conflict of Interest

The authors declare that the research was conducted in the absence of any commercial or financial relationships that could be construed as a potential conflict of interest.

## Publisher’s Note

All claims expressed in this article are solely those of the authors and do not necessarily represent those of their affiliated organizations, or those of the publisher, the editors and the reviewers. Any product that may be evaluated in this article, or claim that may be made by its manufacturer, is not guaranteed or endorsed by the publisher.
